# β-catenin promotes endothelial survival by regulating eNOS activity and flow-dependent anti-apoptotic gene expression

**DOI:** 10.1038/s41419-020-2687-6

**Published:** 2020-06-30

**Authors:** Virginia Tajadura, Marie Haugsten Hansen, Joy Smith, Hannah Charles, Matthew Rickman, Keith Farrell-Dillon, Vasco Claro, Christina Warboys, Albert Ferro

**Affiliations:** 1 School of Cardiovascular Medicine & Sciences, British Heart Foundation Centre of Research Excellence, King’s College London, London, SE1 9NH UK; 2 Department of Bioengineering, Imperial College London, London, SW7 2BP UK; 3 Department of Comparative Biomedical Sciences, Royal Veterinary College, London, NW1 0TU UK

**Keywords:** Cell death, Cell signalling

## Abstract

Increased endothelial cell (EC) apoptosis is associated with the development of atherosclerotic plaques that develop predominantly at sites exposed to disturbed flow (DF). Strategies to promote EC survival may therefore represent a novel therapeutic approach in cardiovascular disease. Nitric oxide (NO) and β-catenin have both been shown to promote cell survival and they interact in ECs as we previously demonstrated. Here we investigated the physiological role of β-catenin as a mediator of NO-induced cell survival in ECs. We found that β-catenin depleted human umbilical vein ECs (HUVEC) stimulated with pharmacological activators of endothelial NO synthase (eNOS) showed a reduction in eNOS phosphorylation (Ser1177) as well as reduced intracellular cyclic guanosine monophosphate levels compared to control cells in static cultures. In addition, β-catenin depletion abrogated the protective effects of the NO donor, S-nitroso-N-acetylpenicillamine, during TNFα- and H_2_O_2_-induced apoptosis. Using an orbital shaker to generate shear stress, we confirmed eNOS and β-catenin interaction in HUVEC exposed to undisturbed flow and DF and showed that β-catenin depletion reduced eNOS phosphorylation. β-catenin depletion promoted apoptosis exclusively in HUVEC exposed to DF as did inhibition of soluble guanylate cyclase (sGC) or β-catenin transcriptional activity. The expression of the pro-survival genes, Bcl-2 and survivin was also reduced following inhibition of β-catenin transcriptional activity, as was the expression of eNOS. In conclusion, our data demonstrate that β-catenin is a positive regulator of eNOS activity and cell survival in human ECs. sGC activity and β-catenin-dependent transcription of Bcl-2, survivin, BIRC3 and eNOS are essential to maintain cell survival in ECs under DF.

## Introduction

Apoptosis is implicated in a number of cardiovascular diseases, in particular the development and progression of atherosclerosis, plaque rupture, ischaemia reperfusion injury and heart failure^1^. Increased endothelial cell (EC) apoptosis is associated with the development of atherosclerotic plaques^2^, vascular injury^3^ and raised vessel permeability^4^. Strategies to promote cell survival may therefore be important in reducing cardiovascular disease.

Atherosclerosis is characterised by dysfunction of the vascular endothelium which is associated with reduced bioavailability and bioactivity of nitric oxide (NO)^5^. Endothelial NO synthase (eNOS), the main vascular source of NO, confers protection from cardiovascular disease^6^, through a number of mechanisms including inhibition of EC apoptosis^7–11^. Similarly, laminar shear stress prevents TNFα and H_2_O_2_-induced apoptosis and this effect is dependent on eNOS activity^9^. NO has previously been shown to inhibit apoptosis at several levels; via S-nitrosylation of caspases^8,12^, increased expression and stability of Bcl-2 by destabilization of MKP-3 mRNA^13^ and inhibition of JNK signalling pathways^7,14^. Recently we identified β-catenin as a novel eNOS binding partner in human umbilical vein EC (HUVEC) and showed that pharmacological activation of eNOS, acting through soluble guanylate cyclase (sGC) and cyclic guanosine monophosphate (cGMP), promotes nuclear translocation of β-catenin and transcription of some β-catenin target genes providing evidence of a novel signalling axis in EC^15^.

β-catenin is a component of the adherens junction, linking VE-cadherin to the actin cytoskeleton, where it plays an important role in the dynamic regulation of endothelial permeability^16^. Non-junctional (cytosolic) β-catenin is rapidly degraded under resting conditions by interacting with an inhibitory complex comprised of APC, axin and GSK3β that phosphorylates β-catenin and targets it for ubiquitin-mediated degradation^16^. Cytosolic β-catenin can be stabilised by canonical and non-canonical Wnt signalling pathways and, as recently established, by NO–cGMP signalling^15^, whereby dephosphorylated (active) β-catenin accumulates and translocates to the nucleus where it regulates gene expression through its interaction with TCF-LEF transcription factors^16^. A subset of β-catenin target genes is associated with anti-apoptotic (cell survival) functions raising the possibility that NO may promote cell survival through transcriptional activation of β-catenin.

Here we sought to assess the role of β-catenin in eNOS signalling and the physiological role of the NO–cGMP–β-catenin axis on apoptosis in EC under disturbed (atheroprone) or undisturbed (atheroprotective) flow.

## Materials and methods

### Cell culture, transfection and application of shear stress

HUVEC were purchased from Promocell as pools from several donors and cultured in Promocell Endothelial Cell Growth Medium (containing 2% FCS). Cells were cultured at 37 °C and 5% CO_2_ and confluent HUVEC at up to passage 6 were used for experiments. Human aortic ECs (HAEC) from single donors were purchased from Promocell and cultured on fibronectin in Endothelial Growth Medium MV (Promocell). HAEC were used up to passage 6. Mouse pulmonary ECs (MPEC) were cultured as previously described^15^. Culture media was supplemented with 100 U/ml penicillin and 100 µg/ml streptomycin. Antibiotic free media was used for the cell assays and added before application of flow (see below) and/or before a treatment was added. At least three different biologically independent cell batches were studied for each experiment.

RNA interference was performed using siRNA sequences specific for human target genes. Non-targeting scrambled sequences were used as a control. HUVEC were transfected with siRNAs (100 nM) using Lipofectamine RNAiMAX (Invitrogen) in serum-free media (Optimem, Invitrogen) without antibiotics. Medium was changed to growth medium 16 h after transfection. Scrambled control and β-catenin siRNA pool were purchased from Ambion and Santa Cruz, respectively.

For assays in which application of flow was required, an orbital shaker was used. EC were seeded at passages 3–7 onto fibronectin-coated six-well plates. Where experiments required immunostaining, EC were seeded onto fibronectin-coated glass-bottom plates (In Vitro Scientific). Once monolayers were confluent (after 24–48 h), media was changed and the six-well plates were placed onto an orbital rotating platform (Grant Instruments) housed inside the incubator and cultured for a further 72 h. The radius of orbit of the orbital shaker was 10 mm and the rotation rate was set to 150 rpm, which caused swirling of the culture medium over the cell surface that creates distinct flow patterns at the centre and edge of the well; cells at the edge are exposed to undisturbed uniaxial flow (UF), whereas cells at the centre are exposed to disturbed multidirectional flow (DF)^17^.

For transfection of HUVEC for flow experiments, immediately prior to seeding into six-well plates, HUVEC were transfected with 100 nM MISSION^®^ pre-designed and validated siRNA targeting β-catenin (Sigma) or 100 nM scrambled control (Ambion) by electroporation. Electroporation was carried out using a Neon™ Transfection System according to manufacturer’s instructions. Cells were seeded at a density of 1 × 10^5^ cells per well and cultured under static conditions for ~6 h to allow cells to adhere and become confluent before exposure to flow using the orbital shaker method. For analysis of protein expression, three wells were pooled for each flow condition. Knockdown of target proteins was confirmed by western blot for each experiment.

EC were exposed to disturbed or UF for 72 h using an orbital shaker (150 rpm; Grant Instruments) housed inside the incubator^17,18^. At least three different biologically independent cell batches were studied for each experiment.

### Cell lysis, fractionation and western blotting

Cells were lysed in ice-cold RIPA buffer (1% Triton X-100, 1% sodium deoxycholate, 2.5 mmol/l ethylenediaminetetraacetic acid (EDTA), 100 mmol/l NaCl, 20 mmol/l Tris-base; pH 7.4) supplemented with protease and phosphatase inhibitor cocktails (Roche). Lysates were incubated on ice for 45 min and then centrifuged for 10 min at 16,000 × *g* to separate soluble from insoluble fractions.

Cell surface and cytosolic extracts were separated from nuclear. Cells were scraped, washed with phosphate-buffered saline (pH 7.4), resuspended in hypotonic buffer (10 mM Hepes (pH 7.9), 1.5 mM MgCl2, 10 mM KCl, 0.2 mM phenylmethylsulfonyl fluoride and 0.5 mM dithiothreitol) and allowed to swell on ice for 10 min. After that 1% NP40 was added and the cells were homogenised with a syringe and needle and vortexed for 2 min. The nuclei were separated by spinning at 3300 g for 5 min at 4 °C. The supernatant was used as soluble cytoplasmic/membrane extract. The nuclear pellet was extracted in nuclear extraction buffer (20 mM Hepes (pH 7.9), 100 mM NaCl, 1.5 mM MgCl2, 1% Triton, 1 mM EDTA, 1 mM EGTA, 10% glycerol, 0.5 % deoxycholate, 0.1% SDS with protease and phosphatase inhibitor cocktails (Roche)) for 30 min on ice and centrifuged at 12,000 × *g* for 30 min. The supernatant was used as a nuclear extract.

Soluble nuclear and cytoplasmic/membrane protein lysates were analysed by SDS-PAGE and immunoblotting. Bound antibodies were visualised with horseradish peroxidase-conjugated anti-IgG antibodies and enhanced chemiluminescence susbtrates (Thermo Scientific). Antibodies for western blotting were obtained from the following sources: anti-cleaved caspase-3, total caspase-3, β-catenin, eNOS, eNOS phosphoS1177, Calnexin, cIAP1, TBP (Cell Signalling), β-catenin, active β-catenin, VE-cadherin, eNOS phosphoS635, eNOS phosphoS114 (BD Biosciences), NOS3, PDHX and GAPDH (Santa Cruz).

### cGMP ELISA

Lysates were extracted by addition of 0.1 mol/L HCl supplemented with 1 mmol/L 3-isobutyl-1-methylxanthine. cGMP concentration was assessed following acetylation using Cyclic GMP EIA Kit (Cayman Chemicals) according to the manufacturer’s instructions. Resulting cGMP concentrations were normalised to protein content per sample.

### Immunostaining and confocal microscopy

HUVEC on fibronectin-coated glass plates (In Vitro Scientific IBL) were fixed with 4% paraformaldehyde solution for 20 min, permeabilized with 0.1% Triton X-100 in PBS for 5 min and blocked with 5% BSA in PBS for a further 30 min. Cells were incubated overnight at 4 °UF C with anti-cleaved caspase-3 antibody (cell signalling), active β-catenin (BD biosciences) or VE-Cadherin (BD Biosciences) or active β-catenin (Millipore) and/or 4′,6-diamidino-2-phenylindole (DAPI) to stain nuclei. Images were generated with a Nikon Spinning disk confocal microscope using a ×20 objective and Nikon software. The percentage of cleaved caspase-3 positive cells was calculated in at least 16 randomly selected fields of view for each condition, covering ~2000–4000 cells to estimate the level of apoptosis.

### En face staining of mouse aortas

All procedures were conducted in accordance with the Directive 2010/63/EU of the European Parliament on the protection of animals used for scientific purposes, as enforced by national legislation, the UK Animal (Scientific Procedures) Act 1986 (as amended), under authorisation of the UK Home Office (Project License No. 70–8934). Male, 8-week-old C57BL/6J mice were purchased from Charles River Laboratories (Harlow, UK), maintained on a 12-hour day/night cycle, and fed a standard breeding/maintenance chow ad libitum (RM3, Special Diets Services, UK) for 2 weeks prior to tissue harvest. Mice were terminally anaesthetised with an overdose of pentobarbitone (120 mg/kg i.p.) and then perfused transcardially with an ice-cold 0.9% saline, 100 U/ml heparin solution, followed by ice-cold 4% paraformaldehyde (Parafix, Pioneer Research Chemicals Ltd, UK). Aortas were removed intact from the heart to the renal bifurcation, further post-fixed in 4% paraformaldehyde at 4 °C overnight, then micro-dissected under a stereomicroscope in 0.1 M phosphate-buffered saline to produce en face preparations. Co-localisation of eNOS (C-20; Santa Cruz) and β-catenin (BD biosciences) was visualised in EC from wild-type C57BL/6 mice by en face staining of susceptible (inner curvature) or protected (outer curvature) regions of the aorta followed by laser scanning confocal microscopy. Nuclei were stained with DAPI.

### Proximity ligation assay (PLA)

PLAs were carried out using a Duolink In Situ Detection Kit as previously described^15^. HUVEC were cultured in six-well plates until confluent and subjected to flow for 72 h using an orbital shaker. Following flow exposure, cells were fixed with 4% paraformaldehyde then permeabilized with 0.5% Triton^®^X-100. In situ PLA was carried out using rabbit anti-eNOS and mouse anti- β-catenin primary antibodies in combination with Duolink In Situ Red Detection Kit (Sigma). PLA was carried out according to the manufacturer’s instructions. Afterwards cells were washed to eliminate excess of reagents and cells were counterstained with DAPI and VE-Cadherin (BD Biosciences) to visualise cell junctions and nuclei, respectively. Images were generated with a Nikon Spinning disk confocal microscope using a ×20 objective and Nikon software NIS elements. PLA analysis was carried out on confocal images. Approximately 2000 cells from four independent experiments were quantified. The PLA signal intensity per cell was analysed using ImageJ and the particle analysis function on PLA images and cells numbers calculated using the same function on DAPI images. A mask for the cell edge was generated with the VE-cadherin image and imposed over the PLA image to measure signal inside and outside of the mask.

### Transferase dUTP nick end labelling (TUNEL) assay

To detect DNA fragmentation in apoptotic cells, terminal TUNEL was performed as previously described^19^. A Click-iT TUNEL Alexa Fluor 594 Imaging Assay was used following the manufacturer instructions (Life Technologies). Briefly cells were fixed using 4% paraformaldehyde in PBS for 15 min and followed by a permeabilization step with 0.25% Triton^®^X-100 for 20 min. Cells were then treated with terminal deoxynucleotidyl transferase for 60 min at 37 °C to allow the incorporation of modified dUTPs at the 3′-OH ends of fragmented DNA, followed by a Click-iT^®^ reaction for 30 min at 37 °C to label ends with a fluorescent dye through click chemistry. Afterwards cells were washed gently to eliminate excess of reagents and cells were counterstained with DAPI and VE-Cadherin (BD Biosciences) to visualise the cell edges and nuclei, respectively. Images were generated with a Nikon Spinning disk confocal microscope using a ×20 objective and Nikon software NIS elements. The percentage of TUNEL positive cells was calculated in 16 selected fields of view for each condition, covering ~2000–4000 cells to estimate the level of apoptosis.

### RNA isolation and quantitative RT-PCR

RNA was isolated from cells using RNeasy Mini kits (Qiagen). Contaminating DNA was removed by on-column DNase digestion (Qiagen). cDNA was prepared using a High-Capacity Reverse Transcription Kit (Thermo Scientific). Quantitative real-time PCR (qPCR) was carried out with cDNA using SYBR green mastermix (Primer Design). GADPH was used as a reference gene. The qPCR oligonucleotide primers used for eNOS were: F: CATCTTCAGCCCCAAACGGA R: AGCGGATTGTAGCCTGGAAC; for Survivin: F: TGAGAACGAGCCAGACTTGG R: TGTTCCTCTATGGGGTCGTCA; for KLF2: F: TGGGCATTTTTGGGCTACCT R: CCCAGTTCCAAGCAACCAGA; for E-SEL: F: GCTCTGCAGCTCGGACAT R: GAAAGTCCAGCTACCAAGGGAAT; for Bcl-2: F: ATGTGTGTGGAGAGCGTCAA R: GGGCCGTACAGTTCCACAAA; for GAPDH: F: CTATAAATTGAGCCCGCAGCC R: ACCAAATCCGTTGACTCCGA; for XIAP: F: AGTGTCTGGTAAGAACTACTG R: CCCATTCGTATAGCTTCTTG; for WISP-1: F: TCATTAAGGCAGGGAAGAAG R: GTCTTAGACTTGTAGGGGATG; for BIRC3: F: ACAAGCAAGAGAACTGATTG R: GATCTGAAACATCTTCTGTGG and for Caspase-3: F: AAAGCACTGGAATGACATC R: CGCATCAATTCCACAATTTC. The amplification process included one cycle of 10 min at 95 °C, 40 cycles for 15 s at 95 °C, followed by 40 cycles for 1 min at 60 °C. Thermal cycling and fluorescence detection were performed using an ABI 7500 Fast Prism (PE Applied Biosystems, Foster City, CA, USA).

### Apoptosis gene expression array

HUVEC were cultured in six-well plates until confluent and subjected to flow for 72 h using an orbital shaker. Following flow exposure, cells were washed twice with cold PBS and total DNA free mRNA was isolated using RNeasy Mini kits (Qiagen) and on-column DNase digestion as described above. mRNA integrity and concentration were determined spectrophotometrically. An RT2 Profiler^tm^ polymerase chain reaction (PCR) apoptosis array (PAHS-012ZA-Qiagen) was performed according to the manufacturer’s instructions. Briefly, a total of 0.5 μg of RNA per sample was used with the RT2 First Strand kit (Qiagen) to obtain cDNA after incubation for 5 min with the gDNA elimination buffer. The PCR amplification process included one cycle of 10 min at 95 °C, 40 cycles for 15 s at 95 °C, followed by 40 cycles for 1 min at 60 °C. Thermal cycling and fluorescence detection were performed using an ABI 7500 Fast Prism (PE Applied Biosystems, Foster City, CA, USA). The signals of the target cDNAs were normalised by comparison with the housekeeping genes HPRT1 supplied within the 96-well microtiter plate. The normalised amount of each target mRNA present in each condition was calculated using a comparative Ct method and using a web-based PCR array data analysis tool (https://www.qiagen.com/gb/shop/genes-and-pathways/data-analysis-center-overview-page/) with 1.5-fold difference set as baseline.

### Statistical analysis

All data are presented as mean ± SEM. Statistical analysis was performed using GraphPad Prism software (v7.1). Statistical significance was assessed using paired two-tailed *t*-test (for comparing two conditions) or by one-way analysis of variance (ANOVA) with repeated measures for multiple conditions. Each *n* is generated with a separate batch of ECs. For HUVEC, these are pools of cells from different donors and for HAEC, in each batch EC had been isolated from a single donor. When using one-way ANOVA, tests for equal variance were run and if significantly different standard deviations were found (thus different variances) in the ANOVA with repeated measures a Gaussian distribution and no sphericity (not equal variability of the differences) were assumed and the Greenhouse–Geisser correction was applied.

## Results

### β-catenin regulates eNOS activity in static HUVEC

**Fig. 1:**
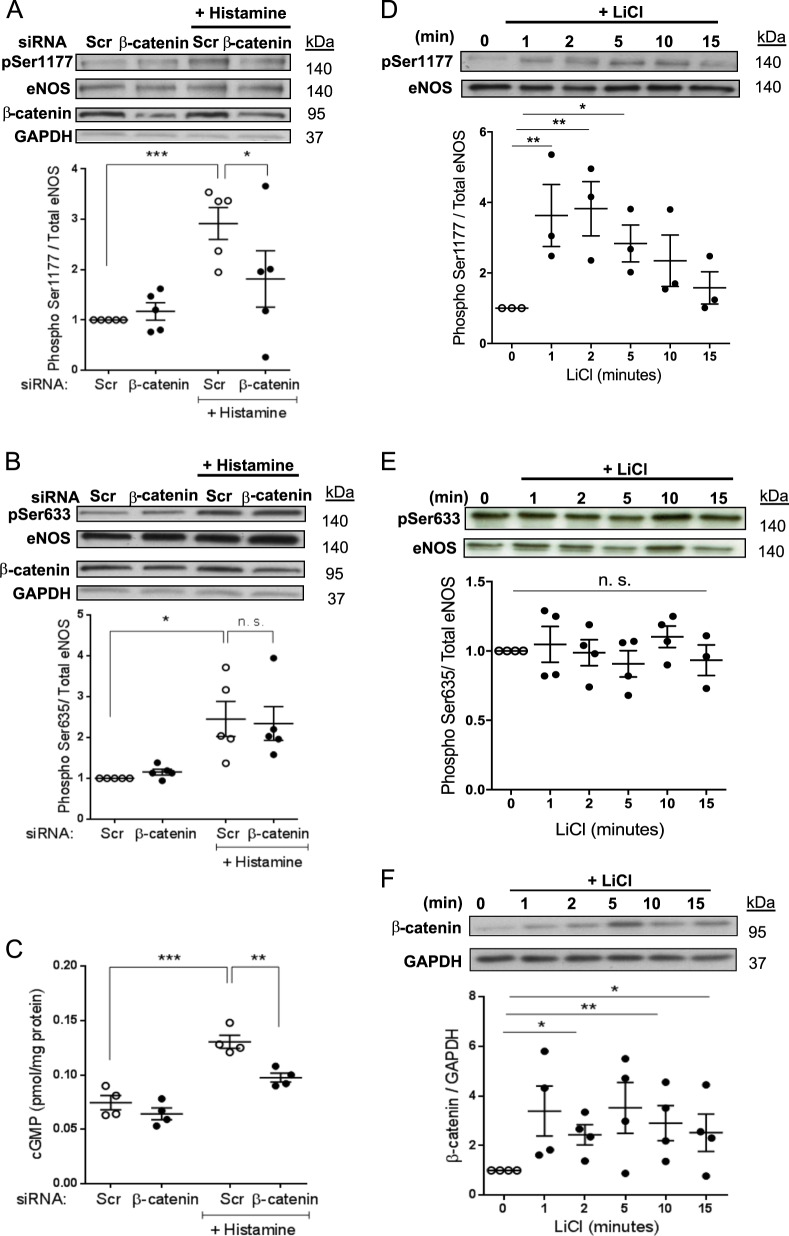
β-catenin depletion inhibits agonist-induced eNOS phosphorylation in static HUVEC. **a**–**c** HUVEC were transfected with siRNA targeting β-catenin (100 nM) or scrambled control siRNA (Scr) and cultured for 72 h before treatment for 5 min with vehicle or histamine (100 mmol/L). Cell lysates were analysed by western blot using total eNOS, b-catenin, phospho-Ser1177 (**a**) or phospho-Ser633 (**b**) antibodies. **c** cGMP levels were quantified by ELISA and results expressed relative to protein content per sample (shown relative to scrambled control; *n* = 4). **d**–**f** HUVEC were cultured for 72 h before treatment for the indicated times with vehicle or LiCl (20 mM). Cell lysates were analysed by western blot using total eNOS, phospho-Ser1177 (**d**) or phospho-Ser633 (**e**) and b-catenin (**f**) antibodies. **a**, **b**, **d**, **e** Results expressed as the densitometric ratio of phospho-eNOS/GAPDH to total eNOS/GAPDH and shown relative to untreated control (*n* = 5); analysis by one-way ANOVA with repeated measures, ns non-significant, *p ≤ 0.05, **p ≤ 0.01, ***p ≤ 0.001.

To investigate whether β-catenin regulates eNOS activation in HUVEC, we assessed eNOS phosphorylation in cells following transfection with a pool of β-catenin siRNA oligonucleotides or an siRNA scrambled control pool. β-catenin depletion did not alter basal levels of eNOS phosphorylation (Figs. 1a, b and S1a, b), however, it did reduce agonist-induced eNOS phosphorylation on Ser1177 (Figs. 1a and S1a). Interestingly, β-catenin depletion had no effect on agonist-induced eNOS phosphorylation on Ser633 (Figs. 1b and S1b). Intracellular cGMP levels are a well-established indicator of bioactive NO levels; we observed that in HUVEC, the histamine-induced increase in cGMP production was reduced following depletion of β-catenin with siRNA (Fig. 1c). The absence of β-catenin protein in HUVEC transfected with β-catenin siRNA was confirmed by western blotting (Fig. S1c), furthermore, eNOS expression was not altered in β-catenin knockdown static cells (Fig. S1d).

To confirm that β-catenin can promote eNOS phosphorylation on Ser1177 and thus increase NO and cGMP production, we treated HUVEC with LiCl that inhibits GSK3β-dependent phosphorylation of β-catenin and promotes its accumulation. We found that elevating β-catenin levels with LiCl increased eNOS phosphorylation on Ser1177 but had no effect on Ser633 in static HUVEC (Fig. 1d–f). Together these data demonstrate that β-catenin enhances agonist-dependent eNOS activation and subsequent cGMP production in HUVEC under static conditions.

### β-catenin mediates the anti-apoptotic effects of NO–cGMP in static HUVEC

**Fig. 2:**
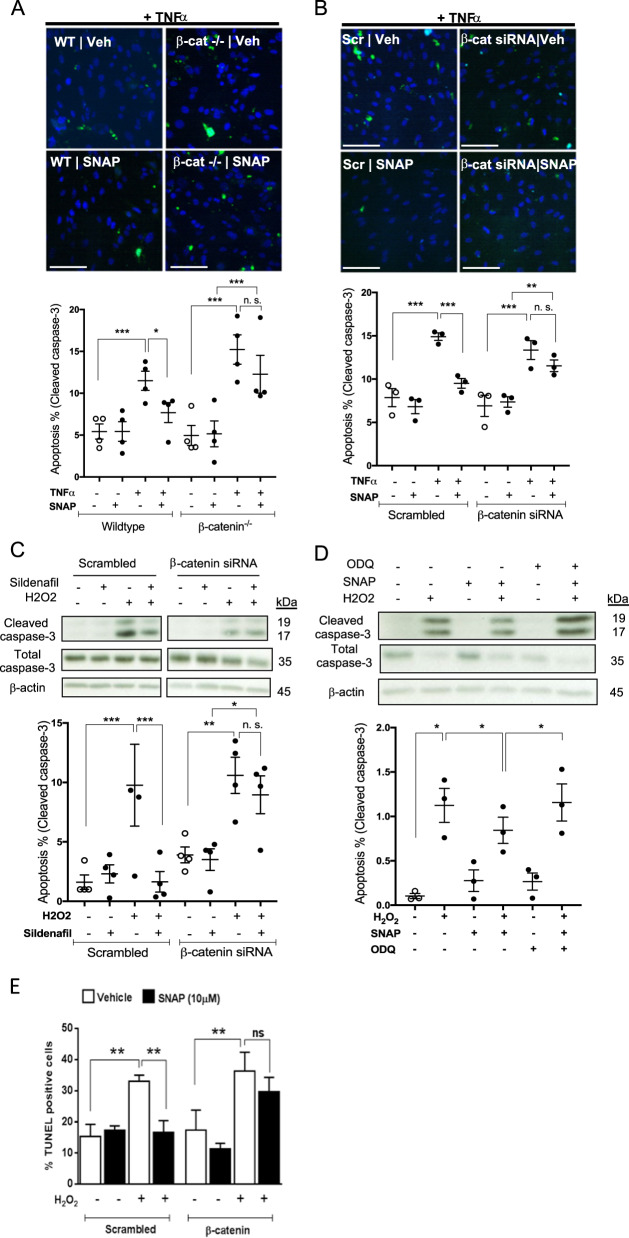
β-catenin mediates the anti-apoptotic effects of NO in static HUVEC. **a** Wild-type and β-catenin^−/−^ MPECs were treated with SNAP (10 µM) or vehicle. **b** HUVEC were transfected with siRNA targeting β-catenin (100 nM) or scrambled control and cultured for 72 h before treatment with TNFα (10 ng/ml) for 18 h in the presence or absence of SNAP (10 µM). **a**, **b** EC were fixed and incubated with anti-cleaved caspase-3 antibody and DAPI. Representative images are shown. The percentage of cleaved caspase-3 positive cells was calculated in five randomly selected fields of view (*n* = 4). **c** HUVEC were transfected with siRNA targeting β-catenin (100 nM) or scrambled control and cultured for 72 h before treatment with H_2_O_2_ (200 µM) for 6 h in the presence or absence of sildenafil (50 nM; *n* = 4). The bands shown by way of example are from the same experiment. **d** HUVEC were treated with H_2_O_2_ (200 µM) for 6 h in the presence of SNAP (10 µM), ODQ (10 µM) or both in combination (*n* = 3). **c**, **d** Cleaved (17 and 19 kDa) and full length (35 kDa) caspase-3 were detected in western blots of cell lysates and normalised to β-actin. Densitometry values are shown expressed relative to vehicle control. **e** HUVEC were transfected with siRNA targeting β-catenin (100 nM) or scrambled control and cultured for 72 h before treatment with H_2_O_2_ (200 µM) for 6 h in the presence or absence of SNAP (10 µM, following which cells were fixed and DNA fragmentation assessed using a Click-IT TUNEL Imaging Kit (*n* = 5). All analyses by one-way ANOVA with repeated measures, ns non-significant, *p ≤ 0.05, **p ≤ 0.01, ***p ≤ 0.001. Scale bars show 100 mm.

Since reduction of β-catenin may impair eNOS function and since NO is an important factor for EC survival^7^, we assessed the potential protective effects of β-catenin, in relation to NO signalling, in static cultures following induction of apoptosis. In wild-type MPEC, application of an NO donor (S-nitroso-N-acetylpenicillamine; SNAP) attenuated TNFα-induced apoptosis, indicated by a lower number of cleaved caspase-3 positive cells in the presence of SNAP, however, in β-catenin^−/−^ MPEC, the protective effects of SNAP on TNFα-induced apoptosis were abrogated (Fig. 2a). We also examined TNFα-induced apoptosis in HUVEC and observed that siRNA depletion of β-catenin reduced the anti-apoptotic action of SNAP in this context (Figs. 2b, e and S1e), demonstrating that β-catenin also mediates the pro-survival effects of NO in human EC under static conditions. Since β-catenin appears to regulate the level of activated eNOS and subsequent cGMP production in HUVEC, we examined activation of caspase-3 by H_2_O_2_ in HUVEC in the presence of sildenafil, a pharmacological sGC activator. Whilst cleaved caspase-3 levels induced by H_2_O_2_ are reduced by sildenafil treatment in scrambled control-treated cells, this was not the case in β-catenin depleted HUVEC (Fig. 2c). Furthermore, ODQ, a pharmacological sGC inhibitor, abrogated the protective effects of SNAP in the presence of H_2_O_2_ (Fig. 2d), indicating that NO exerts its anti-apoptotic effects through sGC activation and cGMP production. Together these data suggest that β-catenin mediates the pro-survival effects of NO and cGMP upon pharmacological (non-physiological) induction of apoptosis in static EC. eNOS expression and activation are known to increase in EC exposed to UF^18^ and enhanced endothelial NO synthesis promotes survival of EC under physiological levels of shear stress against TNFα and H_2_O_2_^8,9^. For these reasons, we next sought to determine whether β-catenin mediates the pro-survival effects of NO under physiological flow conditions.

### β-catenin associates with eNOS in HUVEC exposed to physiological flow

**Fig. 3:**
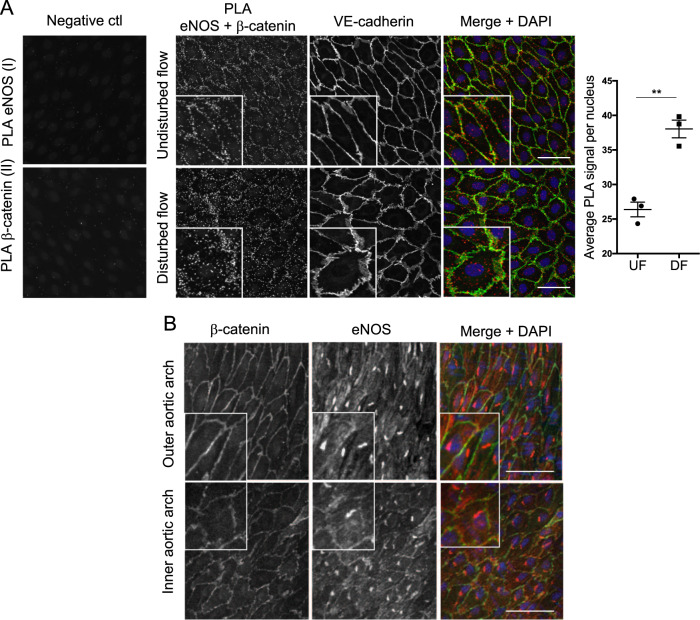
β-catenin interacts with eNOS under flow. **a** HUVEC were exposed to orbital flow for 72 h, fixed and subject to PLA using antibodies targeting eNOS and b-catenin. In control samples, PLA was carried out in the absence of b-catenin (I) or eNOS antibody (II). VE-cadherin and DAPI were used to stain cell junctions and nuclei, respectively. Representative images are shown and the average PLA signal quantified in HUVEC under disturbed flow (DF) or undisturbed flow (UF; *n* = 4); analysis by paired Student’s *t* test, **p ≤ 0.01. **b** EC from protected (outer curvature) or atherosusceptible (inner curvature) regions of the mouse aorta were stained with eNOS (red) and β-catenin (green) antibodies and imaged en face by confocal microscopy. Merged representative images with DAPI (blue) are shown (*n* = 3). Scale bars show 50 mm.

To confirm our previous observation made in static EC^15^, and to test whether eNOS and β-catenin interact under physiological flow conditions, we carried out a PLA in HUVEC subjected to flow for 72 h. We used an orbital shaking platform to generate reproducible spatially separated atheroprotective (undisturbed; uniaxial, high wall shear stress) and atheroprone (disturbed; multiaxial, low wall shear stress) flow patterns^17,18^. As previously described, HUVEC growing under atheroprotective, UF were elongated and aligned meanwhile the cells in the atheroprone, DF central region showed a non-aligned mosaic-like morphology (Figs. 3a and S2a). Furthermore, EC in the UF region expressed high levels of KLF2 and KLF4 and lower levels of MCP-1 and E-selectin compared to EC exposed to DF^20,21^ (Fig. S2b). Co-localisation was observed in EC fixed and stained with anti-eNOS and anti-β-catenin antibodies (Fig. S2a) and amplification products were detected by PLA indicating proximity (<40 nm) of both proteins in HUVEC exposed to both UF and DF (Fig. 3a) but not in controls stained separately with either anti-eNOS or anti-β-catenin antibody. The average PLA signal per cell was lower in EC exposed to UF (Fig. 3a) consistent with increased eNOS activity^15^. β-catenin and eNOS also co-localise in vivo in ECs of the inner and outer curvature of the aortic arch, regions of disturbed and UF, respectively, however, co-localisation was more prominent in the inner aortic arch (Fig. 3b). In contrast with observations made in HUVEC subjected to acute low magnitude shear stress^22^, no difference in the subcellular localisation of β-catenin was observed in HUVEC exposed to chronic flow using an orbital shaker (Fig. S2c).

### β-catenin regulates eNOS phosphorylation in HUVEC exposed to physiological flow

**Fig. 4:**
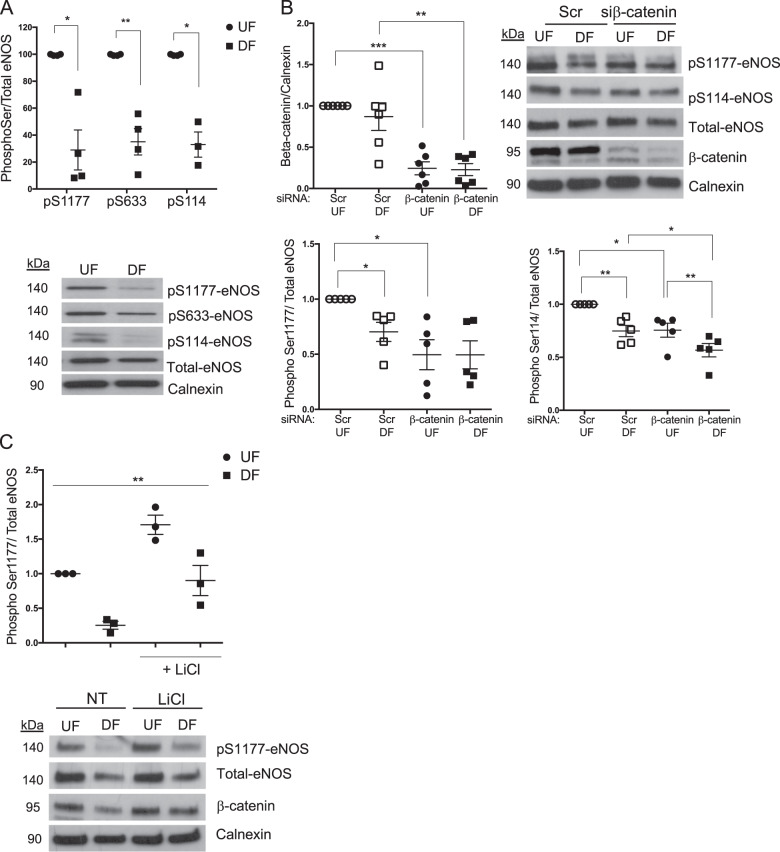
β-catenin regulates eNOS under flow. **a** Cell lysates were obtained from HUVEC exposed to UF or DF for 72 h (*n* = 4). **b** EC transfected with non-targeting scrambled or β-catenin targeting siRNA (100 nM) were exposed to orbital flow for 72 h (*n* = 5). **c** Cell lysates obtained from HUVEC exposed to UF or DF for 72 h and treated with vehicle or LiCl (20 mM) for the last 4 h (*n* = 3). **a**–**c** Cell lysates were analysed by western blot with the indicated antibodies. Results are expressed as the densitometric ratio of phospho-eNOS/eNOS to total eNOS/calnexin and shown relative to levels in UF in vehicle or scr treated conditions; analysis by paired Student’s *t* test (**a**) or analysis by one-way ANOVA with repeated measures (**b**, **c**), *p ≤ 0.05, **p ≤ 0.01, ***p ≤ 0.001.

Since activation of eNOS causes dissociation from β-catenin in static cells^15^, the higher level of interaction observed in EC exposed to DF suggests lower activation of eNOS in these cells. We thus measured eNOS phosphorylation on Ser1177, Ser633 and Ser114 and observed lower eNOS phosphorylation in EC exposed to DF compared to those exposed to UF (Fig. 4a). To investigate whether β-catenin regulates eNOS activation in HUVEC exposed to flow, we assessed eNOS phosphorylation in HUVEC following transfection with β-catenin siRNA or a siRNA scrambled control and exposed to flow for 72 h (Fig. 4b). We observed that eNOS phosphorylation on Ser1177 decreased in EC exposed to UF when β-catenin was depleted but not in EC exposed to DF, consistent with our findings in static EC. eNOS expression was not altered by β-catenin knockdown, compared to scrambled control transfection, although eNOS expression was different in EC exposed to DF compared to UF (Fig. S2d); but interestingly, phosphorylation on Ser114 was reduced in both UF- and DF-exposed HUVEC when β-catenin was depleted (Fig. 4b). In contrast, treatment of HUVEC with LiCl increased Ser1177 phosphorylation in both regions (Fig. 4c), confirming that β-catenin interacts with and can activate eNOS under physiological and atheroprone flow conditions.

### Deletion of β-catenin increases apoptosis only in HUVEC exposed to disturbed flow

**Fig. 5:**
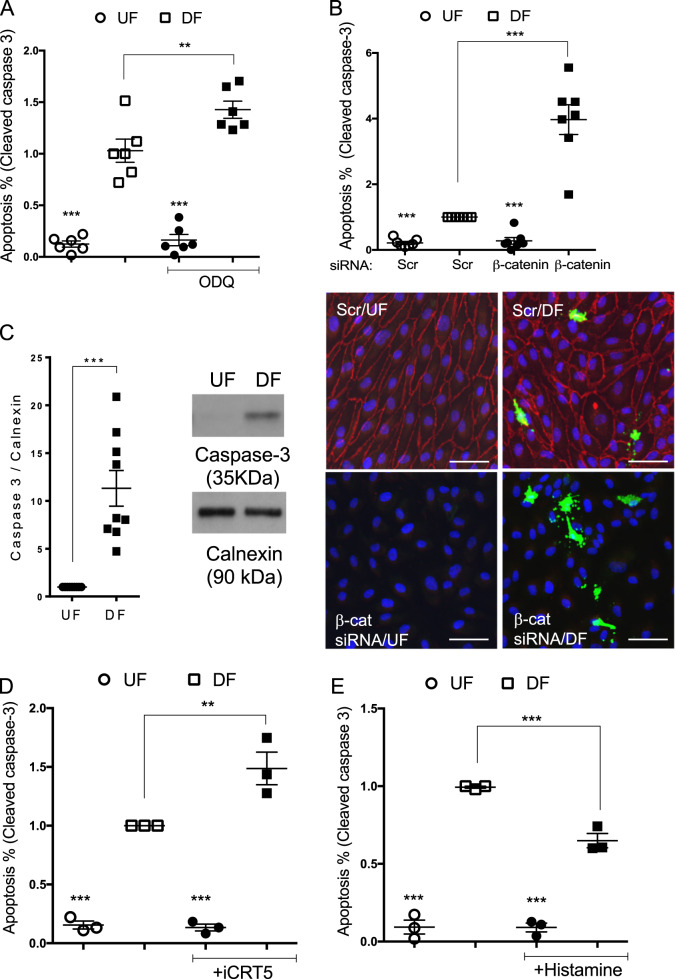
Inhibition of sGC and β-catenin transcriptional activity increases apoptosis in HUVEC exposed to disturbed flow. **a**–**e** HUVEC were exposed to orbital flow for 72 h and treated with DMSO, ODQ (10 mM) (**a**), iCRT5 (50 mM) (**d**) or Histamine (100 mmol/L) (**e**) for the last 24 h of flow exposure or transfected with b-catenin or Scr siRNA (100 nM) (**b**). **b** ECs were fixed and incubated with a cleaved caspase-3 antibody (green), anti-b-catenin (red) and nuclei stained with DAPI. Representative images are shown. **a**, **b**, **d**, **e** The percentage of cleaved caspase-3 positive cells was quantified in regions of undisturbed (UF) or disturbed flow (DF) (*n* = 3–7). **c** HUVEC were exposed to orbital flow or static conditions for 72 h. Full length (35 kDa) caspase-3 was detected in western blots of cell lysates. Results are expressed as the densitometric ratio of caspase-3 to calnexin. Representative western blots are shown (right panel) (*n* = 9); analysis by paired Student’s *t* test **c** or analysis by one-way ANOVA with repeated measures (**a**, **b**, **d**, **e**), *p ≤ 0.05, **p ≤ 0.01, ***p ≤ 0.001. Scale bars show 50 mm.

Atheroprotective, laminar flow activates eNOS and is the predominant source of NO in ECs. It has also been associated with EC survival^8,10^. In contrast, atherogenic shear stress profiles are associated with reduced expression of pro-survival genes and increased apoptosis, both in vitro and in vivo^23,24^. We measured apoptosis by cleaved caspase-3 staining of HUVEC subjected to flow using an orbital shaker and observed that the percentage of apoptosis was higher under DF compared to UF as expected (Fig. S3a). Having established the involvement of β-catenin in NO-mediated anti-apoptotic activity in static cultures, we sought to determine the pro-survival actions of NO and β-catenin under physiological flow conditions. Inhibition of sGC activity with ODQ in HUVEC subjected to shear stress for 72 h, increased apoptosis of HUVEC exposed to DF as assessed by cleaved caspase-3 immunostaining (Fig. 5a). Furthermore, depletion of β-catenin in HUVEC promoted apoptosis exclusively in HUVEC exposed to DF (Figs. 5b and S3b). In agreement with the higher level of apoptotic cells observed in DF-exposed HUVEC, we also detected higher levels of cleaved caspase-3 in lysates from HUVEC subjected to DF for 72 h (Fig. S3c). Unexpectedly, we found that the expression level of pro-caspase-3 (35 kDa) was lower in lysates from EC exposed to UF compared to DF (Fig. 5c), though caspase-3 mRNA expression showed only a 20% increase in cells exposed to DF compared to UF (Fig. S3d), suggesting that a post-translational mechanism may be regulating the protein expression and/or stability of caspase-3 in HUVEC exposed to UF.

### Inhibition of β-catenin transcriptional activity increases apoptosis in HUVEC exposed to disturbed flow

Since β-catenin can regulate the expression of pro-survival genes and has also been shown to act downstream of eNOS/NO/cGMP, we investigated whether inhibiting its transcriptional activity also influenced cell survival under flow conditions. Inhibition of β-catenin-dependent transcriptional activation with the specific inhibitors of β-catenin/TCF-LEF interaction, iCRT5 or FH535^25,26^, also exclusively increased apoptosis in HUVEC under DF in the same conditions (Figs. 5d and S3e) and in HAEC exposed to DF (Fig. S3f). The low level of apoptosis observed under UF was not altered by any method used to manipulate eNOS or β-catenin signalling (Figs. 5a–e and S3e–g). Whilst treatment with histamine, which upregulates eNOS, reduced apoptosis in HUVEC exposed to DF (Fig. 5e), inhibition of eNOS with 100mM L-NAME did not affect apoptosis in HUVEC exposed to either DF or UF (Fig. S3g). These data indicate that signalling through sGC and β-catenin is essential to maintain cell survival in EC exposed to DF and that other additional mechanisms contribute to cell survival in UF-exposed HUVEC.

### Inhibition of β-catenin-dependent transcription downregulates the expression of anti-apoptotic genes in HUVEC under flow

**Fig. 6:**
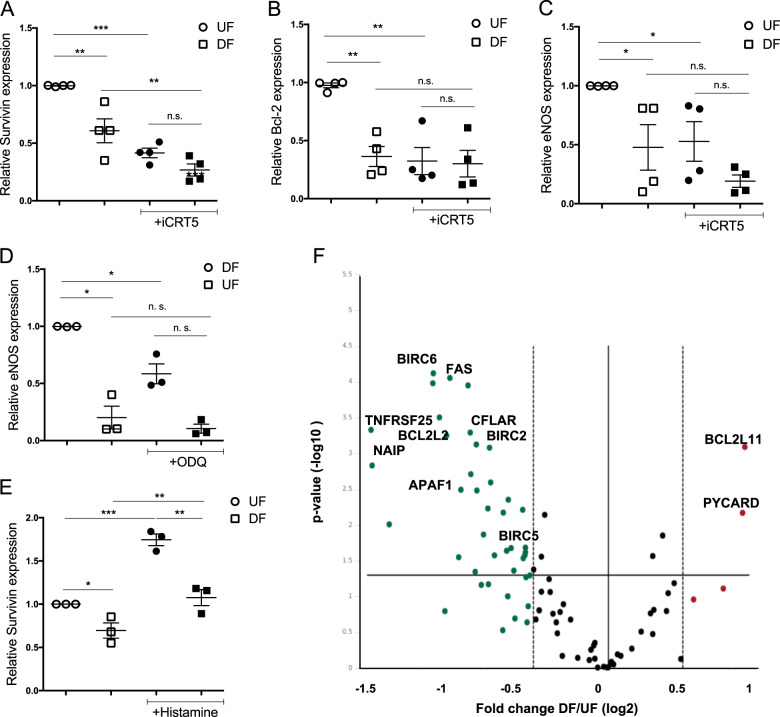
Inhibition of β-catenin downregulates the expression of survivin and Bcl-2 in HUVEC exposed to flow. **a**–**f** HUVEC were exposed to orbital flow for 72 h and treated with DMSO or iCRT5 (50 µM) (**a**–**c**), ODQ (10 mM) (**d**) or histamine (100 mM) (**e**) for the last 24 h of flow or left untreated (**f**). Transcript levels of survivin (**a**, **e**), Bcl-2 (**b**) and eNOS (**c**, **d**) were assessed in HUVEC under disturbed flow (DF) or undisturbed flow (UF) by qRT–PCR using GAPDH as a housekeeping gene. Values are shown relative to expression in vehicle treated HUVEC under UF; analysis by ANOVA with repeated measures (*n* = 3-4); ns not significant, *p ≤ 0.05, **p ≤ 0.01, ***p ≤ 0.001. **f** mRNA samples from HUVEC exposed to DF or UF were analysed using an mRNA expression array targeting 84 apoptotic-related genes. HPRT1 was used as housekeeping gene (*n* = 3). The Volcano plot shows expression fold change of mean across replicates in each condition from DF to UF on *x*-axis (log2) and *p* value (−log10) on *y*-axis. Significantly differentially expressed genes (*p* value < 0.05) are highlighted in red (upregulated; FC > 1.5) or green (downregulated; FC < −1.5). Relevant genes are indicated.

As the inhibition of cGMP signalling and β-catenin-dependent transcription increases apoptosis in HUVEC exposed to DF, we studied the expression of several anti-apoptotic genes that are potential targets of β-catenin: XIAP, WISP-1, Bcl-2 and survivin (BIRC5). Bcl-2 and survivin exhibited differential expression between DF and UF regions in HUVEC (Fig. 6a, b) as did eNOS (Fig. 5c). Interestingly, survivin mRNA expression was reduced when cells were treated with iCRT5 or FH535, in HUVEC exposed to both UF and DF (Figs. 6a and S4a). Bcl-2 and eNOS expression were also reduced in cells under UF in the presence of iCRT5. Treatment of HUVEC with ODQ also reduced the expression of eNOS (Fig. 6d), suggesting the presence of a positive feedback mechanism in the eNOS-sGC-β-catenin pathway. WISP-1 showed very low expression in HUVEC and no differences were found in expression of XIAP between HUVEC exposed to UF and DF in the presence or absence of iCRT5 (not shown).

To identify novel putative genes regulating apoptosis and cell survival in EC exposed to flow, an apoptosis transcriptome array was performed with HUVEC exposed to UF or DF using an orbital shaker for 72 h (Table S1). We classified the genes upregulated and downregulated in DF conditions according to the apoptotic pathways they regulate^27^ and their function as shown in Table 1. We observed that the most significant change in EC exposed to DF was the downregulation of anti-apoptotic genes (Figs. 6f and S5). These include members of the BCL and IAP (inhibitors of apoptosis) families including BIRC5 (survivin), NIAP1, BIRC2 (cIAP1), BIRC6, BCL2L2 and MCL1. Although BIRC3 expression did not change from UF to DF, it was reduced in EC treated with iCRT5 (Fig. S4b) and constitutes a novel target of β-catenin in EC. BIRC2 and BIRC3 are ubiquitin kinases that bind and promote caspase-3 inactivation and degradation^28^. We validated the expression of cIAP1 by western blotting in HUVEC exposed to UF or DF (Fig. S4c) and found its expression upregulated in UF-exposed HUVEC as expected, correlating with low levels of caspase-3 in UF-exposed EC. Treatment of cells with histamine, a pharmacological eNOS activator, that also promotes β-catenin nuclear translocation^15^, reduced apoptosis in HUVEC exposed to DF (Fig. 5e) and increased the level of survivin (Fig. 6e) suggesting a central role for survivin in regulating cell survival in EC. Together these results support the central role of inhibitors of apoptosis, and their positive regulation of expression by β-catenin, in mediating the pro-survival effects of the eNOS–cGMP pathway in EC exposed to flow.

**Table 1:** Apoptosis genes regulated by UF and DF in HUVEC.

Gene names are indicated and common synonyms are shown in brackets. UF and DF represent fold change (2^−ΔΔCt^) in mRNA expression compared to the housekeeping gene (HPRT1) in non-treated HUVEC exposed to UF or DF for 72 h, *n* = 3. Fold regulation is the negative inverse of the fold change. Fold regulation values greater than one indicate upregulation, and fold regulation values less than one indicate downregulation. In red are indicated genes that can behave like anti or pro-apoptotic depending on alternative splicing.

## Discussion

Phosphorylation of eNOS on Ser1177 is associated with increased eNOS activity and we show here in static EC that depletion of β-catenin reduced eNOS phosphorylation on Ser1177 in response to histamine and VEGF stimulation. We also demonstrated increased Ser1177 phosphorylation in EC exposed to UF and found that this was reduced following depletion of β-catenin. Stabilisation of β-catenin in HUVEC treated with LiCl increased Ser1177 phosphorylation in static and flow exposed EC. We conclude that β-catenin activates eNOS in human EC.

We previously reported that eNOS and β-catenin interact in static ECs^15^. We show here that eNOS and β-catenin co-localise in regions of UF and DF in the mouse aorta in vivo with a higher degree of co-localisation in the inner aortic arch region, supporting our finding that eNOS and β-catenin show a higher level of interaction in DF-exposed HUVEC in vitro. This may reflect higher eNOS activation under UF conditions and subsequent dissociation of the complex consistent with our finding that acute pharmacological activation of eNOS causes dissociation from β-catenin^15^. Since reduced levels of β-catenin associate with reduced eNOS phosphorylation in HUVEC, it may be that interaction of eNOS and β-catenin facilitates the access or recruitment of kinases that lead to phosphorylation of eNOS and subsequent dissociation of β-catenin from the complex. PKA and AKT are kinases that phosphorylate Ser1177 and activate eNOS in EC in response to different stimuli such as shear stress or VEGF treatment^29^. PKA and AKT also phosphorylate and stabilise β-catenin and enhance its transcriptional activity^30^. The identification of the putative kinases recruited by β-catenin to eNOS will be an important area of further study in order to understand the mechanism of action for the novel β-catenin-dependent activation of eNOS described here.

We also demonstrate here that β-catenin and sGC signalling are essential mediators of the pro-survival effects of eNOS in static EC challenged with either TNFα or H_2_O_2_ to induce apoptosis. We previously reported that NO/GMP can stabilise β-catenin leading to transcriptional changes^15^. Furthermore, for the first time we present evidence showing that both NO–cGMP signalling and β-catenin transcriptional activity contribute to maintain cell survival in ECs under atheroprone flow conditions. HUVEC treated with the sGC inhibitor ODQ showed an increase in apoptosis in EC exposed to DF that was also observed in both HAEC and HUVEC treated with iCRT5, a specific inhibitor of β-catenin transcriptional activity. Our findings are supported by Saran et al.^31^ who showed that the pro-apoptotic antagonist of Wnt signalling, sFRP4, causes endothelial dysfunction by suppressing NO–cGMP signalling.

Wnt/β-catenin promotes cell survival in static EC^32^, but to our knowledge this is the first study to show that β-catenin regulates apoptosis in EC exposed to atheroprone DF. Several groups have studied the pro-survival and anti-apoptotic effects of UF on EC^8,9^ and identified GSH and NO as key regulators of cell survival in EC under atheroprotective flow conditions. Few studies have assessed the differential expression of the genes and signalling pathways regulating cell survival between disturbed and UF^23,24,33^. Amini et al. demonstrated that JNK activity/expression was required to drive EC apoptosis at atheroprone sites in mice. Similarly, PERP, a p53 regulator was found to be upregulated in atheroprone areas of the porcine aorta, predisposing EC to apoptosis^33^.

S-nitrosylation of β-catenin by iNOS can affect junctional permeability^34^, a characteristic usually associated with EC exposed to DF. Recent findings suggest that β-catenin S-nitrosylation by eNOS impedes binding to the transcription factor TCF4^35^. This seems to be in contrast with our and others’ findings suggesting that NO favours β-catenin separation from VE-cadherin and translocation to the cell nucleus^36^. In addition to NO, ROS production is necessary for S-nitrosylation in cells^37^. ROS increases in EC exposed to DF leading to low NO bioavailability due to the reaction of ROS with NO to form peroxynitrite that promotes protein S-nitrosylation^38^. S-nitrosylation of β-catenin may explain the fact that we observe reduced levels of β-catenin-dependent gene transcription in DF compared to UF-exposed EC. Another interesting possibility that would need further investigation is that S-nitrosylation may allow β-catenin/TCF to discriminate certain promoters or would allow β-catenin interaction with a different member of the TCF family to activate cell survival vs. proliferation programs (Axin2/ cyclinD1 vs. anti-apoptotic genes).

β-catenin controls the transcriptional activation of several pro-survival and anti-apoptotic genes such as WISP-1, Bcl-2 and survivin in static cells^39,40^. Survivin has previously been shown to be downregulated in porcine aortic EC exposed to oscillatory flow^41^ and Bcl-2 has been reported to be downregulated under low shear stress compared to high shear stress in HUVEC. Interestingly, the expression of Bcl-2 in EC under high shear stress depends on eNOS activity^24^. Here we show that in HUVEC, survivin and Bcl-2 are upregulated in EC exposed to UF compared to DF, confirming the previous findings^24,40^, and also that their expression is regulated by β-catenin under flow conditions. In addition, the expression of BIRC3, an inhibitor of apoptosis family member whose expression is flow independent, was also found to be positively regulated by β-catenin in HUVEC. Survivin has been identified as a key mediator of VEGF^40^ and angiopoietin^42,43^ induced survival; since both these stimuli activate eNOS activity their anti-apoptotic actions may be mediated via an NO-driven increase in β-catenin activation and consequent increase in the transcription of pro-survival/anti-apoptotic genes. We also observed that survivin expression increased with histamine treatment in HUVEC. The correlation between survivin expression in EC under flow with NO and β-catenin levels further supports our finding that β-catenin is a novel mediator of the pro-survival effects of NO, possibly via regulation of survivin and Bcl-2 expression. Although our experiments have identified clear changes in survivin and Bcl-2 transcript expression, in future work such changes in expression will need confirmation also at the protein level.

Despite the decreased expression of Bcl-2 and survivin in EC exposed to UF following inhibition of β-catenin, we saw no accompanying increase in apoptosis. This finding suggested that other redundant mechanisms exist in cells exposed to UF that protect the cells from pro-apoptotic stimuli resulting in a powerful pro-survival phenotype. Interestingly we found that the expression of pro-caspase-3 was extremely low in HUVEC exposed to UF when compared to DF conditions. Since caspase-3 is considered the main executioner caspase in EC, this may be a parallel regulatory mechanism limiting apoptosis in EC exposed to UF. Caspases are regulated post-translationally by IAPs (inhibitors of apoptosis), a family of E3 ubiquitin ligases. In particular caspase-3 activity is regulated by BIRC2 (cIAP1) and BIRC3 (cIAP2), that ubiquitinate caspases and promote inactivation and/or subsequent degradation by the proteasome^44^. In HUVEC, cIAP1 which is upregulated by high shear stress was also shown to cause a decrease in caspase-3 activity, although caspase expression was not assessed^45^. Here we describe for the first time that protein levels of caspase-3 are decreased in EC exposed to atheroprotective UF. In our mRNA apoptosis array, BIRC2 (cIAP1) was upregulated under UF compared to DF and protein expression was confirmed by western blotting, suggesting that high levels of cIAP1 in EC exposed to UF may contribute to the degradation and loss of caspase-3 in these cells.

Our data suggest a bidirectional regulation of the eNOS-β-catenin interaction. Upon eNOS activation, β-catenin translocates to the nucleus to activate gene transcription but β-catenin also promotes eNOS activation suggesting that activation of one of the pathways will activate the other one providing a cross talk between the Wnt/β-catenin and NO. In future work, it would be instructive to also investigate the role of caspases-8 and -9, which are upstream of caspase-3, as well as the functional relevance of caspase activation, for example, by the use of pharmacological inhibitors, in the apoptosis pathways investigated here. It will also be important to dissect in further detail the effects on eNOS phosphorylation observed in our study, by assaying individual kinases. Nevertheless, our data provide strong evidence of an interaction between two well-established pro-survival pathways and we propose that the eNOS–cGMP–β-catenin axis is essential to maintain cell survival in EC under DF.

## Supplementary information

Supplementary Figure Legends

Supplementary Figure S1

Supplementary Figure S2

Supplementary Figure S3

Supplementary Figure S4

Supplementary Figure S5

Supplementary Table S1
