# Supplementary material for: β-catenin promotes endothelial survival by regulating eNOS activity and flow-dependent anti-apoptotic gene expression
Source: Cell Death Dis. 2020 Jun 30;11(6):493. doi: 10.1038/s41419-020-2687-6 (PMC7326989; doi:10.1038/s41419-020-2687-6)
Supplement: Supplementary file 1 — Supplementary Figure Legends [file 41419_2020_2687_MOESM1_ESM.docx]

**Supplementary Figure S1: β-catenin depletion inhibits agonist induced eNOS phosphorylation in static HUVEC.** (A-B) HUVEC were transfected with siRNA targeting β-catenin or scrambled control (Scr) and cultured for 72h before treatment for 5 min with vehicle or VEGF (20 nmol/L). Cell lysates were analysed by western blot using anti-β-catenin, total eNOS, phospho-Ser1177 (A) or phospho-Ser633 (B) antibodies. Results expressed as the densitometric ratio of phospho-eNOS/GAPDH to total eNOS/GAPDH (A-B) and shown relative to untreated scrambled control (n=5); analysis by one-way ANOVA with repeated measures, n s: non significant, *p≤0.05, **p≤0.01, ***p≤0.001. (C-E) Cell lysates from non treated HUVEC transfected with siRNA targeting β-catenin or scrambled control (Scr) were analysed by western blot using anti-β-catenin (C,E) total eNOS (D) and GAPDH antibodies and bands quantified by densitometry (n=22); analysis by paired student t-test (C-E) or ANOVA with repeated measures (A-B); n.s., not significant, *p≤0.05, **p≤0.01, ***p≤0.001.

**Supplementary Figure S2: eNOS and β-catenin co-localize in HUVEC.** (A) HUVEC were exposed to orbital flow for 72h, fixed and stained with eNOS (green), β-catenin (red) and DAPI (blue) antibodies. Representative images are shown of HUVEC either in the periphery of the well (Undisturbed flow) or in the centre of the well (Disturbed flow). Scale bar represents 50μm. (B) HUVEC were exposed to orbital flow for 72h and mRNA samples prepared from EC either in the periphery of the well (UF) or in the centre of the well (DF). Transcript levels of KLF2 and E-Selectin (ESEL) were assessed by quantitative RT–PCR using GAPDH as a housekeeping gene (n=3). (C) Lysates from HUVEC exposed to orbital flow for 72h were obtained and separation into a soluble cytoplasm+membrane and nuclear fractions was performed. Lysates were analysed by western blotting using β-catenin, lamin B1 and PECAM1 antibodies. Densitometry values for the cytoplasmic (β-catenin/PECAM-1) and the nuclear (β-catenin/ lamin B1) fractions are shown relative to UF. Representative western blots are shown in right panel (n=4). (D) EC transfected with non-targeting scrambled or β-catenin targeting siRNA (100 nM) were exposed to orbital flow for 72h. Lysates obtained from UF and DF exposed cells were analysed by western blotting, (n=5). Analysis by paired Student’s t–test (B-C) or analysis by ANOVA with repeated measures (D), n s: non significant, *p≤0.05, **p≤0.01.

**Supplementary Figure S3: β-catenin regulates apoptosis in EC exposed to flow.** (A) HUVEC were exposed to orbital flow for 72h, fixed and stained with cleaved caspase 3 (green), active β-catenin (red) and DAPI (blue) antibodies. Representative images are shown of HUVEC either in the periphery of the well (UF) or in the centre of the well (DF). Scale bar shows 25μm. (B) EC transfected with non-targeting scrambled or β-catenin targeting siRNA (100 nM) were exposed to orbital flow and cell lysates analysed by western blot (n=6). (C) Lysates from HUVEC exposed to UF or DF for 72h were immunoblotted with caspase 3 and calnexin antibodies. Densitometry values are shown relative to UF. Representative western blots with calnexin and cleaved caspase 3 (17kDa) are shown in right panel (n=3). (D) HUVEC were exposed to orbital flow for 72h and mRNA samples prepared from EC either in the periphery of the well (UF) or in the centre of the well (DF). Transcript levels of caspase 3 were assessed by quantitative RT–PCR using GAPDH as a housekeeping gene (n=3). (E) HUVEC were exposed to orbital flow for 72h and treated with FH535 (50 μM) or DMSO for the last 24h. EC were fixed and incubated with an antibody that detects the cleaved form of caspase-3 (Asp175) and nuclei stained with DAPI. The percentage of cleaved caspase-3 positive cells was calculated in the periphery of the well (UF) and in the centre of the cell (DF). Values are shown relative to vehicle treated DF (n=3). (F) HAEC were exposed to orbital flow for 72h and treated with iCRT5 (50 μM) or DMSO for the last 24h. The relative rate of apoptosis was quantified as in D (n=5). (G) HUVEC were exposed to orbital flow for 72h and treated with L-NAME (100 µM) or left untreated for the last 24h and the relative rate of apoptosis was quantified as in D (n=4); analysis by paired Student’s t–test (B-D) or ANOVA with repeated measures (E-G); n s: non-significant, *p≤0.05, **p≤0.01, ***p≤0.001.

**Supplementary Figure S4: Inhibitors of apoptosis are downregulated in DF exposed HUVEC.** (A) HUVEC were exposed to orbital flow for 72h and treated with FH535 (25 μM) or DMSO for the last 24h. Transcript levels of survivin were assessed by quantitative RT–PCR using GAPDH as a housekeeping gene. Values are shown relative to DMSO (n=3). (B) HUVEC were exposed to orbital flow for 72h and treated with iCRT5 (50 μM) or DMSO for the last 24h. Transcript levels of BIRC3 were assessed as in B (n=3). (C) HUVEC were exposed to orbital flow for 72h and protein levels of BIRC2 were assessed in HUVEC under disturbed flow (DF) or undisturbed flow (UF) by western blot. Values are shown relative to expression in EC under UF. Right panel shows blot representative of n=3; analysis by paired Student’s t–test (A,C) or one-way ANOVA with repeated measures (B); n s: non significant, *p≤0.05, ***p≤0.001).

**Supplementary Figure S5: Anti-apoptotic genes are down-regulated in EC exposed to disturbed flow.** HUVEC were exposed to orbital flow for 72h and mRNA samples prepared from EC either in the periphery of the well (UF) or in the centre of the well (DF). An mRNA expression array targeting 84 apoptotic related genes was performed. HPRT1 was used as housekeeping gene (n=3). Significantly differentially expressed genes with a p-value < 0.05 and with a FC>1.5 or FC<-1.5 were selected as hits. (A) 39% of the genes tested (33 out of 84) showed at least a 1.5-fold change in expression between DF and UF conditions. Of those, 31 were genes downregulated in EC exposed to DF compared to UF and only 2 genes were found to be upregulated. Of the 84 genes tested, 19 encoded death receptors or ligands, 19 encoded anti-apoptotic or pro-survival proteins, 27 encoded pro-apoptotic factors, 15 encoded effector caspases and other downstream executor proteins [27] and 4 encoded proteins that can be pro- or anti-apoptotic (Supplementary Table S1). Both of the genes that were upregulated in DF conditions, encoded pro-apoptotic proteins. Of the genes found to be downregulated, 35.4% (11 out of 31) encoded anti-apoptotic or pro-survival proteins, 22.5% (7 out of 31) pro-apoptotic proteins, 22.5% (7 out of 31) death receptors and 18% (6 out of 31) caspases and other executor proteins. The anti-apoptotic genes downregulated in EC exposed to DF constitute 57.8% of the anti-apoptotic genes included in the array (11 out of 19) meanwhile the other genes represented 26.9%, 36.8 % and 33% of the pro-apoptotic, receptors and ligands or effector genes tested respectively. (B) Pie chart showing the number of hits in each category.

**Supplementary Table S1: Summary of apoptosis-related gene expression in HUVEC exposed to UF and DF.**
