# Supplementary figures and images for: β-catenin promotes endothelial survival by regulating eNOS activity and flow-dependent anti-apoptotic gene expression
Source: Cell Death Dis. 2020 Jun 30;11(6):493. doi: 10.1038/s41419-020-2687-6 (PMC7326989; doi:10.1038/s41419-020-2687-6)

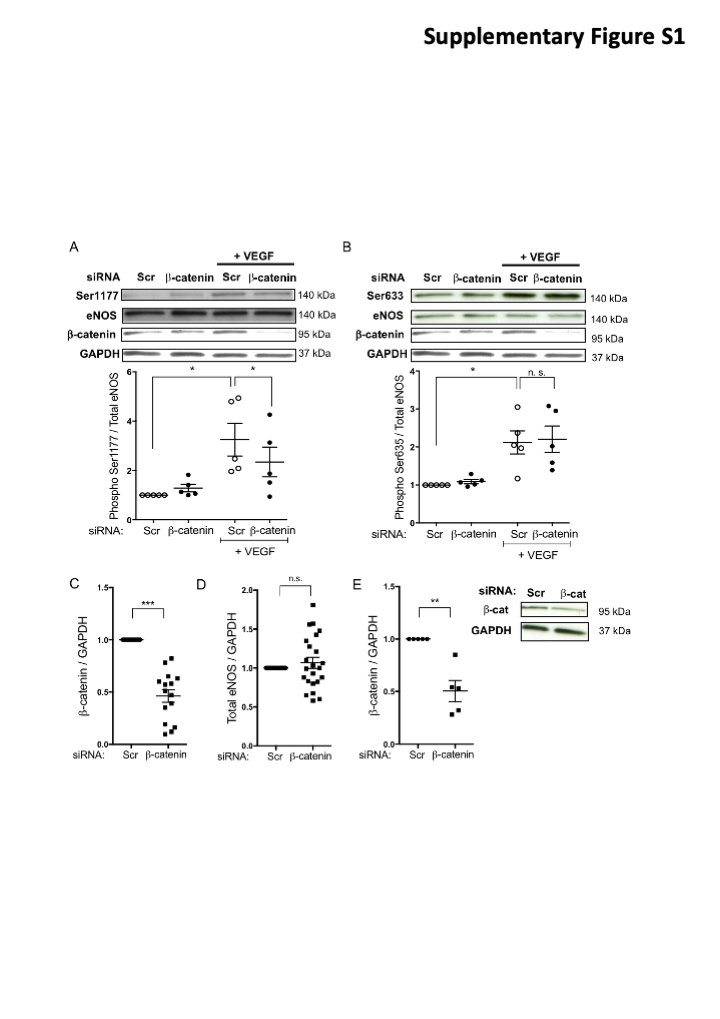

Supplement: Supplementary file 2 — Supplementary Figure S1 [file 41419_2020_2687_MOESM2_ESM.tif]

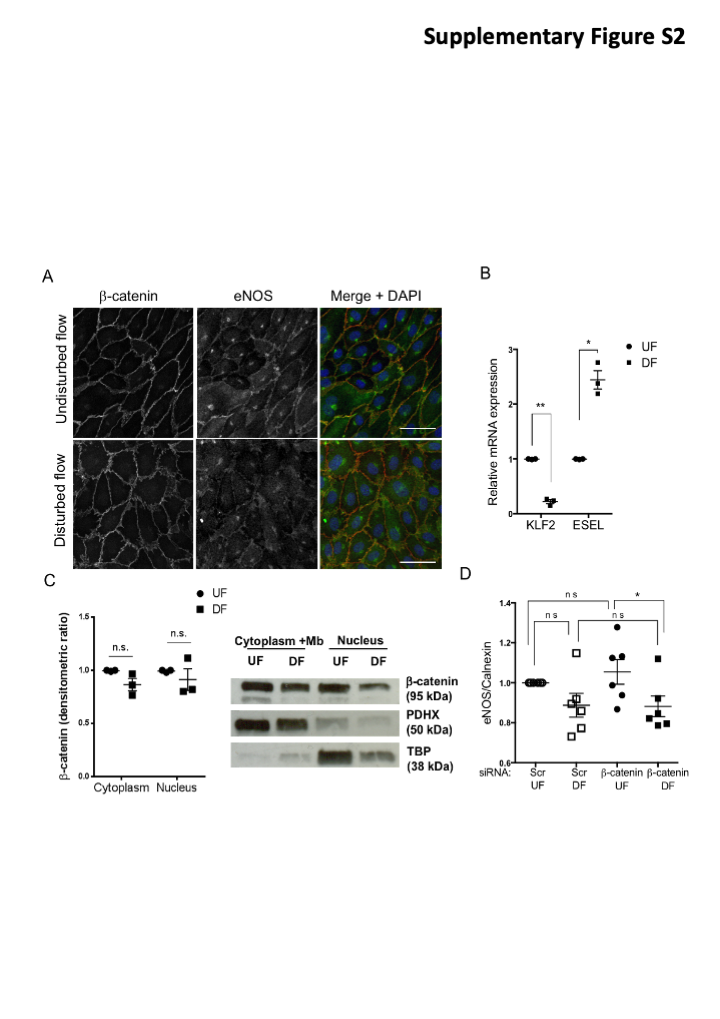

Supplement: Supplementary file 3 — Supplementary Figure S2 [file 41419_2020_2687_MOESM3_ESM.tif]

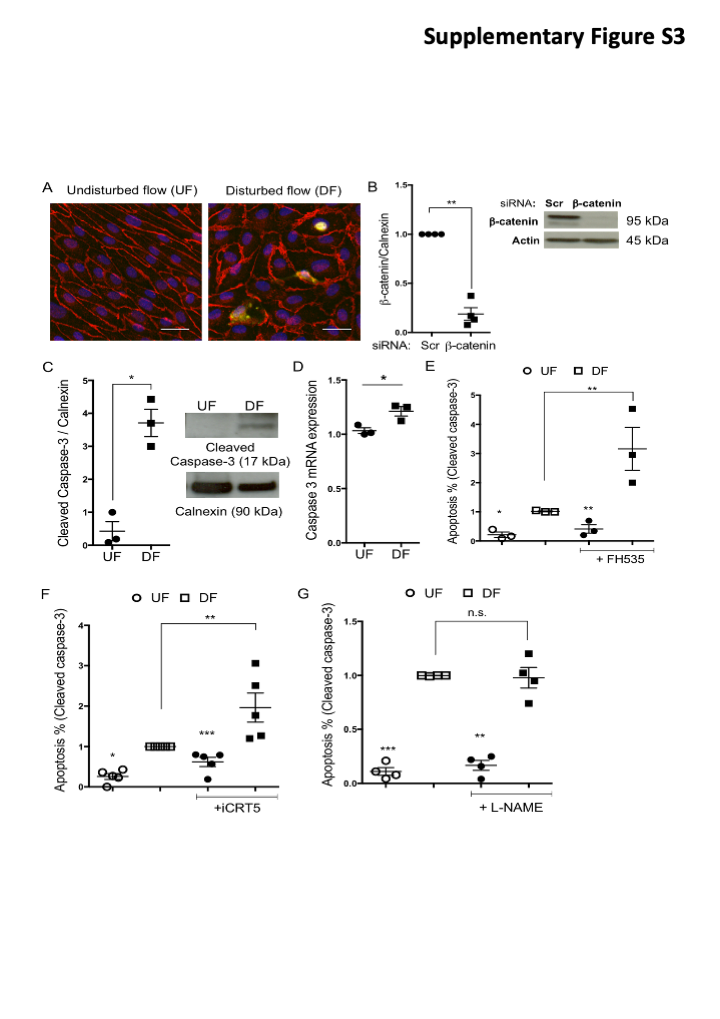

Supplement: Supplementary file 4 — Supplementary Figure S3 [file 41419_2020_2687_MOESM4_ESM.tif]

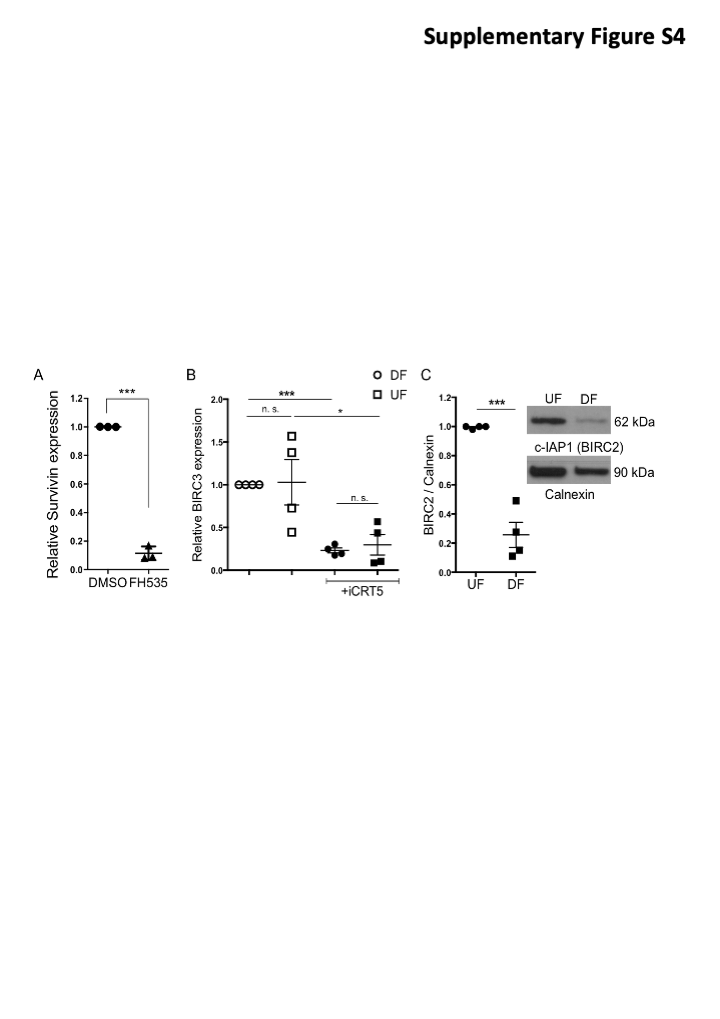

Supplement: Supplementary file 5 — Supplementary Figure S4 [file 41419_2020_2687_MOESM5_ESM.tif]

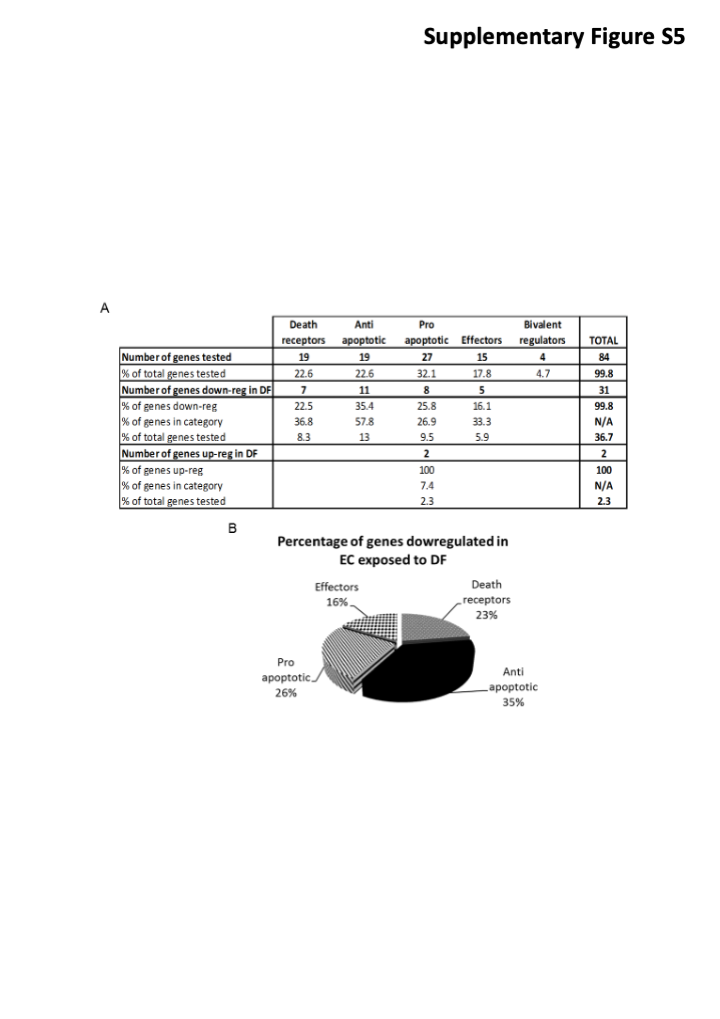

Supplement: Supplementary file 6 — Supplementary Figure S5 [file 41419_2020_2687_MOESM6_ESM.tif]
