# Supplementary material for: β-catenin promotes endothelial survival by regulating eNOS activity and flow-dependent anti-apoptotic gene expression
Source: Cell Death Dis. 2020 Jun 30;11(6):493. doi: 10.1038/s41419-020-2687-6 (PMC7326989; doi:10.1038/s41419-020-2687-6)
Supplement: Supplementary file 7 — Supplementary Table S1 [file 41419_2020_2687_MOESM7_ESM.docx]

|  | | | | | | |
| --- | --- | --- | --- | --- | --- | --- |
|  |  | |  |  | **Fold Regulation** | ***p-value*** |
| **Position** | **Refseq** | | **Symbol** | **Apoptotic pathway/category** | **DF vs UF** | ***DF vs UF*** |
| A01 | NM_005157 | | ABL1 | Pro-apoptotic | -1.5651 | *0.02068* |
| A02 | NM_004208 | | AIFM1 | Pro-apoptotic | -1.8943 | *0.002535* |
| A03 | NM_005163 | | AKT1 | Anti-apoptotic/pro-survival | 1.1985 | *0.308315* |
| A04 | NM_001160 | | APAF1 | Pro-apoptotic | -2.223 | *0.003211* |
| A05 | NM_004322 | | BAD | Pro-apoptotic | 1.3863 | *0.089515* |
| A06 | NM_004323 | | BAG1 | Anti-apoptotic/pro-survival | -2.2514 | *0.028103* |
| A07 | NM_004281 | | BAG3 | Anti-apoptotic/pro-survival | -1.4116 | *0.007195* |
| A08 | NM_001188 | | BAK1 | Pro-apoptotic | -1.5654 | *0.023977* |
| A09 | NM_004324 | | BAX | Pro-apoptotic | -1.0736 | *0.736664* |
| A10 | NM_003921 | | BCL10 | Pro-apoptotic | 1.0144 | *0.868758* |
| A11 | NM_000633 | | BCL2 | Anti-apoptotic/pro-survival | -1.3674 | *0.086221* |
| A12 | NM_004049 | | BCL2A1 | Anti-apoptotic/pro-survival | -2.4236 | *0.159624* |
| B01 | NM_138578 | | BCL2L1 | Anti-apoptotic/pro-survival | 1.001 | *0.972438* |
| B02 | NM_020396 | | BCL2L10 | Pro-apoptotic | -1.1049 | *0.768924* |
| B03 | NM_006538 | | BCL2L11 | Pro-apoptotic | 2.1026 | *0.000809* |
| B04 | NM_004050 | | BCL2L2 | Anti-apoptotic/pro-survival | -2.4041 | *0.000559* |
| B05 | NM_016561 | | BFAR | Bifunctional (Pro/Anti-apoptotic) | -1.2742 | *0.127776* |
| B06 | NM_001196 | | BID | Pro-apoptotic | 1.0543 | *0.641333* |
| B07 | NM_001197 | | BIK | Pro-apoptotic | -2.107 | *0.001943* |
| B08 | NM_001166 | | BIRC2 | Anti-apoptotic/pro-survival | -2.043 | *0.000747* |
| B09 | NM_001165 | | BIRC3 | Anti-apoptotic/pro-survival | -1.0248 | *0.952439* |
| B10 | NM_001168 | | BIRC5 | Anti-apoptotic/pro-survival | -1.6939 | *0.020966* |
| B11 | NM_016252 | | BIRC6 | Anti-apoptotic/pro-survival | -2.5799 | *0.000076* |
| B12 | NM_004330 | | BNIP2 | Bifunctional (Pro/Anti-apoptotic) | -1.3498 | *0.173635* |
| C01 | NM_004052 | | BNIP3 | Bifunctional (Pro/Anti-apoptotic) | 1.2745 | *0.027125* |
| C02 | NM_004331 | | BNIP3L | Bifunctional (Pro/Anti-apoptotic) | 1.2849 | *0.153039* |
| C03 | NM_004333 | | BRAF | Pro-apoptotic | -2.591 | *0.000104* |
| C04 | NM_033292 | | CASP1 | Executor and effector proteins | -1.226 | *0.209515* |
| C05 | NM_001230 | | CASP10 | Executor and effector proteins | -1.9028 | *0.000829* |
| C06 | NM_012114 | | CASP14 | Executor and effector proteins | -1.5276 | *0.050478* |
| C07 | NM_032982 | | CASP2 | Executor and effector proteins | -1.4381 | *0.085554* |
| C08 | NM_004346 | | CASP3 | Executor and effector proteins | -1.5627 | *0.053641* |
| C09 | NM_001225 | | CASP4 | Executor and effector proteins | -1.0731 | *0.442365* |
| C10 | NM_004347 | | CASP5 | Executor and effector proteins | -1.7717 | *0.295184* |
| C11 | NM_032992 | | CASP6 | Executor and effector proteins | -1.095 | *0.552437* |
| C12 | NM_001227 | | CASP7 | Executor and effector proteins | 1.0731 | *0.67144* |
| D01 | NM_001228 | | CASP8 | Executor and effector proteins | -1.7663 | *0.006703* |
| D02 | NM_001229 | | CASP9 | Executor and effector proteins | -1.7186 | *0.004419* |
|  |  | |  |  | **Fold Regulation** | ***p-value*** |
| **Position** | **Refseq** | | **Symbol** | **Apoptotic pathway/category** | **DF vs UF** | ***DF vs UF*** |
| D03 | NM_001242 | | CD27 | Death receptors and ligands | -3.2806 | *0.009785* |
| D04 | NM_001250 | | CD40 | Death receptors and ligands | -1.316 | *0.325528* |
| D05 | NM_000074 | | CD40LG | Death receptors and ligands | -1.9686 | *0.013581* |
| D06 | NM_001252 | | CD70 | Death receptors and ligands | -1.658 | *0.202356* |
| D07 | NM_003879 | | CFLAR | Anti-apoptotic/pro-survival | -2.1141 | *0.00051* |
| D08 | NM_001279 | | CIDEA | Executor and effector proteins | -1.5513 | *0.228807* |
| D09 | NM_014430 | | CIDEB | Executor and effector proteins | -1.7336 | *0.02284* |
| D10 | NM_003805 | | CRADD | Pro-apoptotic | -1.0064 | *0.972725* |
| D11 | NM_018947 | | CYCS | Pro-apoptotic | -2.4964 | *0.000313* |
| D12 | NM_004938 | | DAPK1 | Pro-apoptotic | -1.9165 | *0.067307* |
| E01 | NM_004401 | | DFFA | Executor and effector proteins | 1.0199 | *0.811724* |
| E02 | NM_019887 | | DIABLO | Pro-apoptotic | -1.3226 | *0.228869* |
| E03 | NM_003824 | | FADD | Pro-apoptotic | 1.0296 | *0.882835* |
| E04 | NM_000043 | | FAS | Death receptors and ligands | -2.3589 | *0.000088* |
| E05 | NM_000639 | | FASLG | Death receptors and ligands | -2.0387 | *0.003289* |
| E06 | NM_001924 | | GADD45A | Pro-apoptotic | 1.1384 | *0.534175* |
| E07 | NM_003806 | | HRK | Pro-apoptotic | -1.4549 | *0.155279* |
| E08 | NM_000875 | | IGF1R | Anti-apoptotic/pro-survival | -1.2957 | *0.163936* |
| E09 | NM_000572 | | IL10 | Anti-apoptotic/pro-survival | -2.0572 | *0.044915* |
| E10 | NM_000595 | | LTA | Death receptors and ligands | -1.0591 | *0.978922* |
| E11 | NM_002342 | | LTBR | Death receptors and ligands | -1.3791 | *0.057066* |
| E12 | NM_021960 | | MCL1 | Anti-apoptotic/pro-survival | -1.5719 | *0.026338* |
| F01 | NM_004536 | | NAIP | Anti-apoptotic/pro-survival | -3.6072 | *0.00147* |
| F02 | NM_003998 | | NFKB1 | Anti-apoptotic/pro-survival | -1.438 | *0.027655* |
| F03 | NM_006092 | | NOD1 | Executor and effector proteins | -2.137 | *0.000112* |
| F04 | NM_003946 | | NOL3 | Anti-apoptotic/pro-survival | -1.5877 | *0.006106* |
| F05 | NM_013258 | | PYCARD | Pro-apoptotic | 2.0788 | *0.006742* |
| F06 | NM_003821 | | RIPK2 | Pro-apoptotic | 1.3458 | *0.01402* |
| F07 | NM_000594 | | TNF | Death receptors and ligands | -1.4549 | *0.155279* |
| F08 | NM_003844 | | TNFRSF10A | Death receptors and ligands | -1.7219 | *0.099106* |
| F09 | NM_003842 | | TNFRSF10B | Death receptors and ligands | -1.6676 | *0.043374* |
| F10 | NM_002546 | | TNFRSF11B | Death receptors and ligands | -1.4796 | *0.208317* |
| F11 | NM_001065 | | TNFRSF1A | Death receptors and ligands | -1.8542 | *0.02651* |
| F12 | NM_001066 | | TNFRSF1B | Death receptors and ligands | -1.1768 | *0.715645* |
| G01 | NM_014452 | | TNFRSF21 | Death receptors and ligands | 1.8737 | *0.07722* |
| G02 | NM_003790 | | TNFRSF25 | Death receptors and ligands | -3.6224 | *0.000468* |
| G03 | NM_001561 | | TNFRSF9 | Death receptors and ligands | -1.2813 | *0.674319* |
| G04 | NM_003810 | | TNFSF10 | Death receptors and ligands | 1.2602 | *0.171339* |
| G05 | NM_001244 | | TNFSF8 | Death receptors and ligands | -1.5442 | *0.136328* |
| G06 | NM_000546 | | TP53 | Pro-apoptotic | 1.3765 | *0.159191* |
| G07 | NM_005426 | | TP53BP2 | Pro-apoptotic | -1.9209 | *0.005864* |
| G08 | NM_005427 | | TP73 | Pro-apoptotic | -1.9914 | *0.068535* |
|  |  | |  |  | **Fold Regulation** | ***p-value*** |
| **Position** | **Refseq** | | **Symbol** | **Apoptotic pathway/category** | **DF vs UF** | ***DF vs UF*** |
| G09 | NM_003789 | | TRADD | Pro-apoptotic | -1.4995 | *0.041893* |
| G10 | NM_021138 | | TRAF2 | Pro-apoptotic | -1.5841 | *0.029047* |
| G11 | NM_003300 | | TRAF3 | Pro-apoptotic | -1.0815 | *0.481774* |
| G12 | NM_001167 | | XIAP | Anti-apoptotic/pro-survival | 1.4862 | *0.744222* |
| H01 | NM_001101 | | ACTB | N/A | 1.5942 | *0.109598* |
| H02 | NM_004048 | | B2M | N/A | 1.4349 | *0.065041* |
| H03 | NM_002046 | | GAPDH | N/A | 1.2743 | *0.3344* |
| **H04** | **NM_000194** | | **HPRT1** | **N/A** | **1** | ***0*** |
| H05 | NM_001002 | | RPLP0 | N/A | 1.6461 | *0.013995* |
| ***Bold: Housekeeping gene used for quantification** | | | | |  |  |
|  | |  |  |  |  |  |
